# Early Clinical and Inflammatory Predictors of Supplemental Oxygen Requirement in Children Hospitalized with Acute Lower Respiratory Infection: A Multivariable Analysis

**DOI:** 10.3390/microorganisms14091926

**Published:** 2026-09-01

**Authors:** Klaudia Grudzińska, Kacper Bułdyś, Cezary Dubaj, Ernest Kuchar, Anna Piwowarczyk

**Affiliations:** 1 Department of Pediatrics with Clinical Assessment Unit, Medical University of Warsaw, 02-091 Warsaw, Poland; klaudia.grudzinska@uckwum.pl (K.G.); anna.piwowarczyk@wum.edu.pl (A.P.); 2 Department and Clinic of Hematology, Cellular Therapies and Internal Medicine, Wroclaw Medical University, 50-367 Wroclaw, Poland; 3 Department of Non-Surgical Sciences, Faculty of Medical and Health Sciences, Kazimierz Pułaski University of Radom, 26-600 Radom, Poland

**Keywords:** patient acuity, prognosis, oximetry, respiratory sounds, logistic models

## Abstract

Early identification of children at risk of severe acute respiratory infection remains a clinical challenge. Although C-reactive protein (CRP), procalcitonin (PCT), and white blood cell (WBC) count are commonly assessed at hospital admission, their predictive value for severity assessment remains uncertain. We aimed to evaluate the utility of clinical features, a respiratory multiplex PCR test detecting 23 pathogens, imaging findings, and early inflammatory biomarkers in predicting subsequent supplemental oxygen requirement, used as a marker of severe acute lower respiratory infection (ALRI), among hospitalized children. The primary outcome was the need for supplemental oxygen during hospitalization. We conducted a single-center retrospective cohort study of children admitted to the pediatric department between May 2022 and June 2023 with ALRI or other conditions for which respiratory infection was considered but subsequently excluded. All included patients underwent multiplex respiratory PCR testing. We included 439 children in the analysis [median age, 20 mo (IQR: 5–54); 52.7% males]. Out of them, 178 (40.5%) required supplemental oxygen. The strongest positive association with subsequent supplemental oxygen requirement was observed for low oxygen saturation on admission [OR 8.83 (95% CI, 3.00–26.02; *p* < 0.001)], although this variable was documented in only about half of the cohort (52.4%). The strongest predictors of subsequent supplemental oxygen requirement in multivariable analysis were age, abnormal auscultatory findings, and the cumulative number of danger signs, including hypoxemia, tachypnea, dyspnea, and increased work of breathing. Among the 23 pathogens tested, respiratory syncytial virus (RSV) was the only pathogen consistently associated with a more severe course: 60.3% of RSV-positive children required supplemental oxygen, compared with 36.9% of RSV-negative children (OR 2.59, 95% CI 1.53–4.40; *p* < 0.001). For predicting subsequent supplemental oxygen requirement, no single marker had strong utility. These findings support the importance of structured bedside clinical assessment for identifying hospitalized children who may subsequently require supplemental oxygen. Given incomplete documentation of some vital signs, associations involving these variables should be interpreted with caution.

## 1. Introduction

Acute lower respiratory infections (ALRI) are one of the most serious public health problems in the global pediatric population, responsible for 13.1% of all under-5 mortality worldwide [1,2]. Although mortality is concentrated predominantly in low- and middle-income countries, these infections also constitute a substantial clinical burden in high-income settings, where the incidence rate among children under 5 was estimated at 1114 per 100,000 in 2021, and respiratory infections remain one of the leading causes of pediatric hospitalization [3,4]. Among the etiological factors, Streptococcus pneumoniae remains the leading bacterial cause of death from ALRI in children under 5 years of age [1]. Respiratory syncytial virus (RSV) is the principal viral pathogen responsible for severe ALRI in young children, with infants under 6 months disproportionately affected [5]. Although recently introduced immunoprophylactic strategies have shown high success in reducing RSV-related hospitalizations in high-income settings, severe RSV disease still poses a significant burden globally [6,7]. Despite substantial advances in the prevention and treatment of pediatric respiratory infections, severe disease remains a major clinical challenge, underscoring the need for novel tools for early risk stratification and identification of risk factors for severe outcomes [1,2]. Numerous prognostic tools have been developed and validated to assess the severity of community-acquired pneumonia (CAP) in adults. Among the most widely used are the Pneumonia Severity Index (PSI) and the Confusion, Urea, Respiratory Rate, Blood Pressure, and Age ≥65 years (CURB-65) score. Both have been shown to accurately predict mortality risk and to support clinical decision-making regarding the appropriate site of care [8,9]. In the pediatric population, however, validated prognostic tools remain limited, largely because many of the variables included in adult severity scores are not directly applicable to children [10,11]. Although several pediatric severity assessment scores for community-acquired pneumonia have been developed (Table S1), validation studies show unclear value in identifying patients at risk of severe disease and adverse clinical outcomes [12,13,14,15,16]. Additionally, no single sign reliably identifies patients at risk of severe ALRI [13,14,17,18]. Multivariate prognostic models that integrate multiple clinical parameters, such as hypoxemia, increased respiratory effort, and tachypnea, are more effective at distinguishing between children with mild and severe disease [19]. These observations are reinforced by large-cohort data showing that only hypoxemia and increased work of breathing reliably predict severe outcomes as single clinical signs [17]. Laboratory markers, although traditionally used, show modest performance in differentiating severity. C-reactive protein (CRP) and procalcitonin (PCT) are the most extensively studied biomarkers for severity stratification in pediatric CAP. However, current evidence suggests that neither marker alone is sufficiently accurate to reliably differentiate between severe and mild disease, although emerging data suggest that elevated levels of both markers may help identify children at risk of the most severe complications, including empyema and sepsis [20,21]. At the same time, increasing attention has been directed toward novel biomarkers, including soluble triggering receptor expressed on myeloid cells-1 (sTREM-1), angiopoietin-2 and routinely available hematological parameters such as the white blood cell (WBC) count and the neutrophil-to-lymphocyte ratio (NLR), which have shown promising prognostic potential in recent studies [22,23,24,25]. In parallel with the assessment of the host inflammatory response, identification of the causative pathogen itself plays an increasingly important role in severity stratification. Studies have demonstrated that the type of pathogen detected, particularly RSV and influenza viruses, is more strongly associated with disease severity than the presence of co-infection [26]. Furthermore, in infants under 1 year of age, detection of two or more viruses was associated with a significantly more severe disease course [27]. These outcomes suggest that pathogen identification may be a valuable adjunct to clinical assessment and inflammatory biomarkers for risk stratification of pediatric CAP [26,27]. Multiplex PCR panels enable detection of dozens of respiratory pathogens with substantially higher sensitivity and faster turnaround than standard methods, facilitating earlier targeted therapy and antibiotic de-escalation in children with pneumonia [28,29,30,31,32]. Despite extensive research, there remain critical knowledge gaps in prognostication for children with severe ALRI. This study attempts to determine whether combining clinical and radiologic assessment, respiratory PCR results, and inflammatory biomarkers on admission improves the stratification of risk for severe respiratory disease in hospitalized children. The results may aid decision-making regarding triage and treatment, as well as identify markers for targeted monitoring or intervention.

## 2. Materials and Methods

### 2.1. Study Design, Setting, and Participants

We conducted a single-center retrospective cohort study at the Department of Pediatrics of Masovian Specialist Hospital in Radom, Poland, where approximately 1232 children are admitted each year. The Department of Pediatrics is a tertiary-level pediatric unit without a pediatric intensive care unit (PICU). On-site respiratory support is limited to supplemental oxygen, including low-flow devices and high-flow nasal cannula. Children requiring intensive care-level support, including invasive or non-invasive mechanical ventilation, are stabilized and transferred to a center with a PICU; such decisions are made on an individual clinical basis rather than according to a fixed protocol. We identified children younger than 18 years of age admitted from the ED to inpatient care who underwent PCR-based respiratory panel testing for 23 respiratory pathogens between May 2022 and June 2023. Eligible participants either presented with symptoms of a respiratory tract infection or underwent respiratory PCR panel testing to exclude the respiratory tract as the source of infection. In most patients admitted to the hospital, a complete blood count, routine biochemical tests, and CRP measurements were performed as part of standard clinical care. During the study period, PCT testing was not routinely performed. Patients who did not undergo respiratory multiplex PCR testing were excluded from the study. Chest imaging (radiography and/or lung ultrasound) was performed on a case-by-case basis at the clinician’s discretion in patients with suspected CAP or to identify the source of fever/infection. Institutional protocols for the diagnosis and management of community-acquired pneumonia were consistent with current clinical guidelines.

### 2.2. Data Collection and Variables

Data were collected from electronic medical records. The unit of analysis was the hospitalization episode rather than the child. Of the 439 episodes, 22 were repeat admissions, corresponding to 20 children with more than one hospitalization (18 children with two episodes and two children with three); the 439 episodes therefore originated from 417 individual children. This number differs from the 17 rehospitalizations reported as a secondary outcome, which were restricted to readmissions occurring within 30 days of discharge. Study investigators (K.G. and K.B.) separately reviewed patients’ electronic medical records and collected demographic information (age and sex) and clinical data. The data extracted included medical history, vital signs, characteristics of dyspnea, diagnosis at admission to the ward using ICD-10 codes and text within the admission summary, length of stay (LOS) in hospital, and need for rehospitalization. In addition, we documented respiratory support and treatments, and collected laboratory and imaging reports. Chest imaging (radiography and/or lung ultrasound) was classified as abnormal when any consolidation, interstitial infiltrate, bronchopneumonic change, or pleural abnormality was reported. Respiratory pathogens were identified using a respiratory PCR panel that detects 23 viral and bacterial respiratory pathogens (BioFire Respiratory 2.1 Plus Panel, BioFire Diagnostics, LLC, Salt Lake City, UT, USA). All patient records and information were anonymized and de-identified before analysis. Any disagreements were discussed by two researchers (A.P. and K.G.) until an agreement was reached.

### 2.3. Definitions and Outcome Measures

Pneumonia was defined or redefined if it was the treating physician’s admission diagnosis, based on clinical and radiologic findings. Disease severity was classified according to a previously published classification system, which defines moderate-severe disease as hospitalization during which at least one of the following occurred: administration of at least one intravenous (IV) fluid bolus, continuous IV fluid therapy for more than 12 h, supplemental oxygen therapy, escalation of antibiotic treatment from an aminopenicillin to an antibiotic from another class, complicated pneumonia (moderate-to-large pleural effusion, metastatic infection associated with pneumonia, lung abscess, or pulmonary necrosis), or presumed sepsis, defined as systemic inflammatory response syndrome requiring antibiotic therapy and administration of an IV fluid bolus of ≥40 mL/kg.

Although this composite classification was used to define disease severity, the primary outcome analyzed in this study was the requirement for supplemental oxygen during hospitalization, which served as the operational marker of severe disease. Supplemental oxygen requirement was selected as the sole analyzed outcome because it was a clinically relevant and consistently documented component of the composite severity classification, whereas the remaining components were infrequent, limiting reliable analysis of the full composite outcome. Pleural effusion measuring 10–20 mm was also considered a criterion for severe disease; however, all three patients with pleural effusion simultaneously required supplemental oxygen. Therefore, for the purposes of statistical analysis, these patients were included in a single outcome group defined by the requirement for supplemental oxygen.

Potential clinical predictors of severe disease were selected based on previously published danger signs reported in the literature. Owing to the retrospective study design, only variables available and reliably documented in the medical records were included in the analysis. The evaluated danger signs included: (1) accessory respiratory muscle use; (2) audible wheezing without auscultation; (3) dyspnea; (4) reduced appetite, feeding refusal or feeding difficulty; (5) fever at admission; (6) grunting; (7) peripheral oxygen saturation ≤ 92%; (8) tachypnea; and (9) vomiting [13,14,18,20,33,34,35,36]. Secondary outcomes were length of hospital stay (days) and rehospitalization.

All danger signs were assessed at the time of hospital admission based on the initial clinical evaluation and documented medical records. In contrast, the requirement for supplemental oxygen was assessed throughout the entire hospitalization and was recorded if oxygen therapy was initiated at any time during the hospital stay.

### 2.4. Statistical Analysis

All statistical analyses were performed using R software, version 4.3.3 (R Foundation for Statistical Computing, Vienna, Austria).

Continuous variables were presented as the median and interquartile range, and categorical variables as frequency and percentage. Comparisons between groups were performed using the Wilcoxon test for continuous variables and Fisher’s exact test for categorical variables. The association between potential predictors and the need for oxygen therapy was assessed using logistic regression. In univariate analyses, each predictor was evaluated separately. Standard logistic regression was used for univariable analyses; for binary predictors with zero cells indicating potential complete or quasi-complete separation, mean bias-reduced logistic regression was applied, while estimates based on very sparse data or yielding unstable or non-convergent models were reported as not estimable. Prior to modeling, data completeness, predictor distributions, outliers, the linearity of continuous variables with the endpoint logit, and multicollinearity were assessed. Linearity was assessed by comparing linear models with models using natural spline functions. Multicollinearity was assessed based on Spearman’s correlation coefficient and VIF or adjusted GVIF values. Multivariate models were built sequentially by adding successive groups of predictors. Models were compared using AIC, BIC, AUC, the Brier score, and likelihood ratio tests. The selection of the final model took into account predictive quality, simplicity, availability of variables at the time of admission, and clinical rationale. Discriminatory power was assessed based on the AUC. The optimism-adjusted AUC, Brier score, intercept, and calibration slope were calculated. A two-sided significance level of 0.05 was adopted. Exploratory analyses were interpreted without correction for multiple comparisons.

An AI-based language assistant (OpenEvidence, https://www.openevidence.com/, accessed on 20 April 2026) was used during the preparation of this manuscript to support the literature review, language editing, and the design ofTable 1. All AI-generated content was critically reviewed, verified, and revised by the authors, who take full responsibility for the accuracy and integrity of the final manuscript.

## 3. Results

### 3.1. Cohort Characteristics

**Figure 1:**
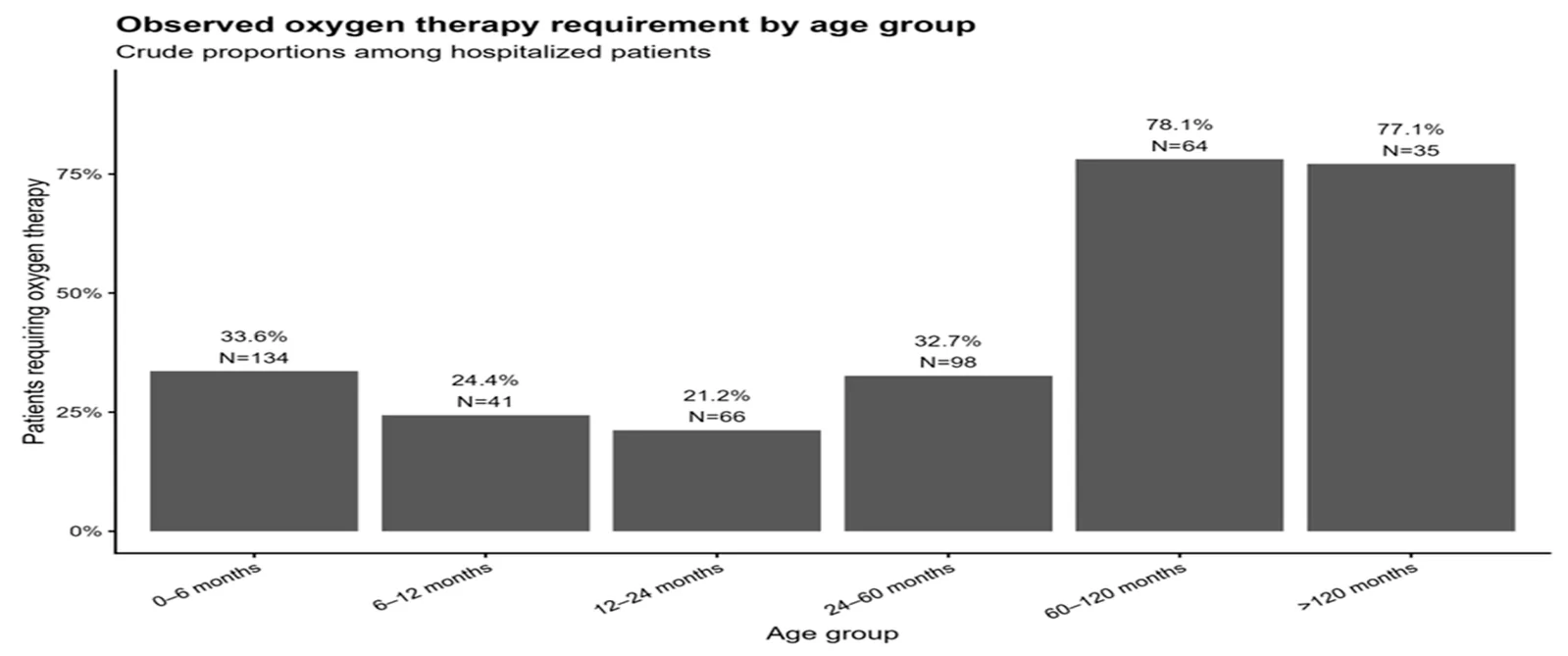
Observed oxygen therapy requirement by age group.

Of 439 children, 231 (52.7%, one missing value) were male, and the median age was 20 months (IQR 5–54). The highest number of hospitalizations occurred during the winter season (146/439, 33.3%), followed by the autumn season (124/439, 28.2%). A respiratory pathogen was detected in 353 (80.4%) cases, and 113 (25.7%) had a viral co-infection. Pneumonia was diagnosed in 75 (17.1%) patients. Supplemental oxygen was required by 178 children (40.5%) (Figure 1). Children requiring supplemental oxygen had a significantly higher median age (Table 1) and presented more frequently with features of respiratory compromise/distress. Significant clinical predictors of the need for oxygen included the use of accessory respiratory muscles, dyspnea, lower peripheral oxygen saturation (≤92%), tachypnea, and the number of danger signs. Abnormal lung auscultation findings, including crackles, prolonged expiration, and wheezing, as well as the diagnosis of pneumonia, were also associated with oxygen requirement. Among additional variables, consolidation detected on lung ultrasound and an increased neutrophil-to-lymphocyte ratio were significantly associated with the need for supplemental oxygen.

### 3.2. Impact of Candidate Predictors on Subsequent Supplemental Oxygen Requirement-Univariable Associations

Crude associations for all candidate predictors are shown in Table 2. Age was significantly associated with oxygen supplementation, OR 1.02 (95% CI 1.01–1.02; *p* < 0.001). The strongest positive association with oxygen supplementation was observed for peripheral oxygen saturation on admission (one of the evaluated danger signs), OR 8.83 (95% CI, 3.00–26.02; *p* < 0.001); this variable was documented in only 230/439 children (52.4%) and is also a direct determinant of the clinical decision to initiate oxygen therapy, so the estimate should be interpreted with particular caution. Among the remaining evaluated danger signs odds ratio for accessory respiratory muscle use was 4.31 (95% CI 2.45–7.57; *p* < 0.001), dyspnea 4.52 (95% CI 2.75–7.44; *p* < 0.001), tachypnea 6.23 (95% CI 3.36–11.54; *p* < 0.001) were associated with oxygen supplementation, as was the number of danger signs present at admission 1.59 (95% CI 1.35–1.88; *p* < 0.001). Additional clinical features showing associations included abnormal lung auscultation with OR 3.74 (95% CI 2.49–5.62; *p*< 0.001), presence/suspicion of chronic disease 2.31 (95% CI 1.33–4.02; *p* = 0.003), cough 1.92 (95% CI 1.29–2.84; *p* = 0.001), max body temperature 0.67 (95% CI 0.51–0.88; *p* = 0.004; n = 288), crackles 6.44 (95% CI 3.54–11.72; *p* < 0.001), diagnosis of pneumonia 3.20 (95% CI 1.91–5.37; *p* < 0.001), prolonged expiration 4.16 (95% CI 2.20–7.86; *p* < 0.001), rhonchi 1.76 (95% CI 1.11–2.79; *p* = 0.017), wheezing on auscultation 2.69 (95% CI 1.54–4.72; *p* < 0.001). The presence of bacterial superinfection among children with viral infection was associated with an OR of 3.31 (95% CI 1.06–10.41; *p* = 0.040). However, viral co-infection did not show a significant association. Analysis of laboratory parameters identified NLR with OR 1.12 (95% CI 1.06–1.19; *p* < 0.001) as a predictor of oxygen requirement. In contrast, CRP, PCT, and WBC did not show an association with the outcome. Of the identified pathogens, Adenovirus [OR 0.50 (95% CI 0.29–0.84; *p* = 0.009)], RSV [OR 2.59 (95% CI 1.53–4.40; *p* < 0.001)], SARS-CoV-2 C [OR 0.27 (95% CI 0.1–0.73; *p* = 0.009)] showed an association with oxygen requirement. Radiological findings were subsequently analyzed. Pleural effusion on chest radiography [OR 4.53 (95% CI 1.21–16.97; *p* = 0.025)] and consolidation on lung ultrasound [OR 4.77 (95% CI 2.18–10.45; *p* < 0.001)] were more frequently observed in children requiring supplemental oxygen.

### 3.3. Multivariable Model

**Figure 2:**
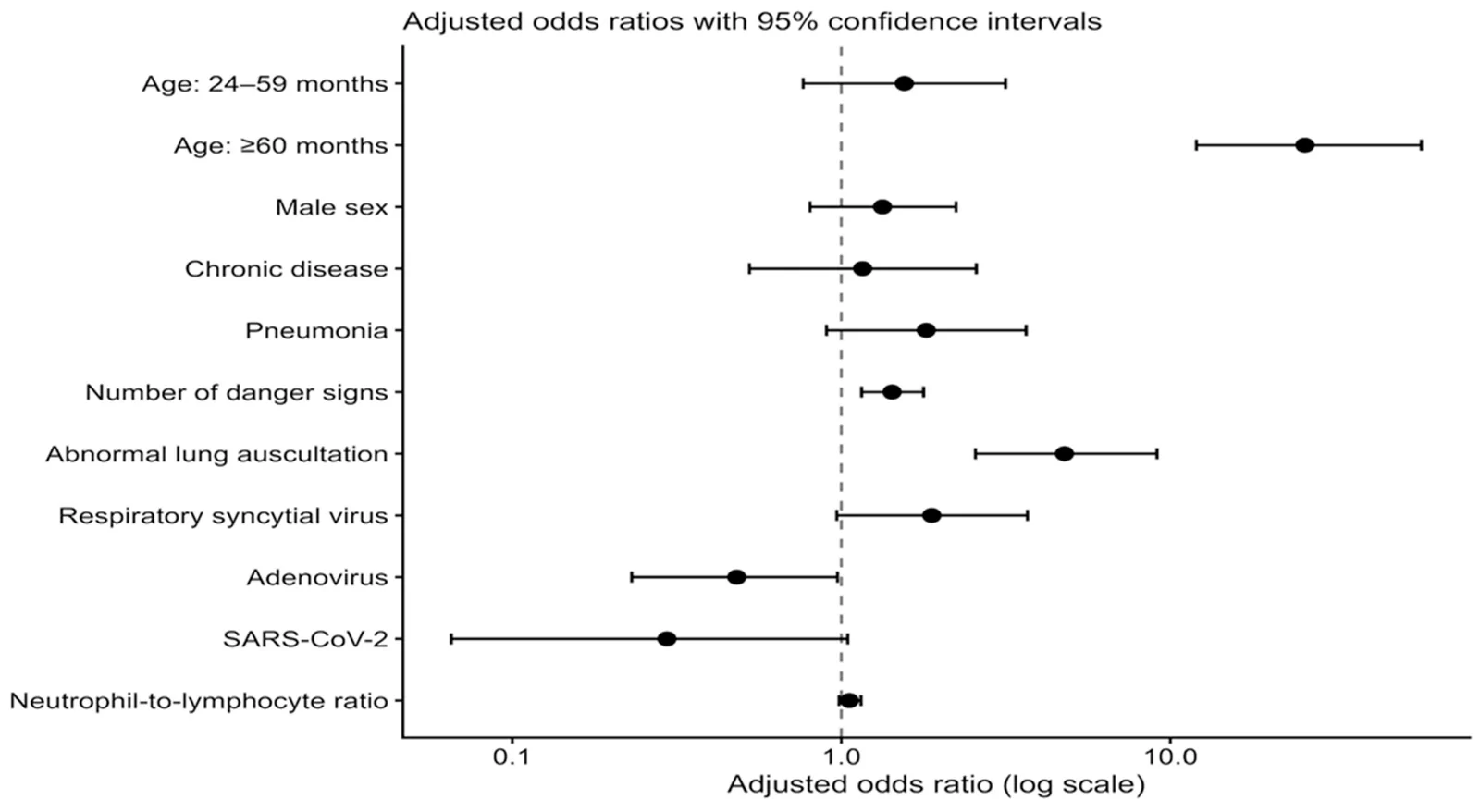
Final multivariable prediction model.

Based on the comparison of competing multivariable models, the final prediction model was developed using complete-case data from 429 children (429/439, 97.7%), including 175 patients who required supplemental oxygen. The model included age category, sex, chronic disease or suspected chronic condition, pneumonia, the number of danger signs, abnormal lung auscultation, RSV, adenovirus, SARS-CoV-2, and NLR (Figure 2). Among all candidate predictors, age ≥60 months, the number of danger signs, and abnormal lung auscultation showed the strongest adjusted associations with the requirement for oxygen therapy. Children aged ≥60 months had substantially higher odds of requiring oxygen therapy than those younger than 24 months (adjusted OR 25.7, 95% CI 12.0–57.9); however, the width of this confidence interval indicates considerable imprecision, and the magnitude of this estimate should not be regarded as a transportable effect size. No significant difference was observed between children aged 24–59 months and those younger than 24 months. Abnormal lung auscultation (adjusted OR 4.77, 95% CI 2.56–9.11) and an increasing number of danger signs (adjusted OR 1.43 per additional sign, 95% CI 1.15–1.78) remained independently associated with oxygen therapy after adjustment for the remaining predictors. Pneumonia and RSV showed positive associations with oxygen therapy, although their confidence intervals included the null value. Adenovirus was associated with lower odds of oxygen therapy, whereas SARS-CoV-2 showed a similar trend with wider confidence intervals. The NLR showed only a modest adjusted association after accounting for the remaining predictors. The final model demonstrated good apparent discrimination (AUC 0.865), which remained high after internal bootstrap validation (optimism-corrected AUC 0.850). Calibration was also satisfactory, with an optimism-corrected calibration slope of 0.901 and a corrected Brier score of 0.154.

### 3.4. Discriminative Performance of Inflammatory Markers

The key negative finding of this study is that conventional inflammatory markers do not discriminate between children who require oxygen and those who do not. CRP (AUC 0.526), PCT (AUC 0.518), and WBC (AUC 0.509) performed no better than chance. The NLR was the best-performing marker (AUC 0.586) and discriminated better than CRP, PCT, WBC, the neutrophil and lymphocyte counts.

### 3.5. Pathogens and Secondary Outcomes

A respiratory pathogen was detected in 80.4% of children. Human Rhinovirus/Enterovirus was the most common detection (38.7%), followed by adenovirus (18.9%) and RSV (15.5%). Among the 23 respiratory pathogens assessed, RSV showed the strongest association with supplemental oxygen requirement: 41 of 68 RSV-positive children (60.3%) required oxygen compared with 137 of 371 RSV-negative children (36.9%), corresponding to an absolute difference of 23.4 percentage points (OR 2.59, 95% CI 1.53–4.40; *p* < 0.001). Adenovirus (*p* = 0.009), SARS-CoV-2 (*p* = 0.006), and Coronavirus NL63 (*p* = 0.032) were also associated with the requirement for supplemental oxygen (Table 3). *p* values in Table 3 were obtained with Fisher’s exact test and therefore differ slightly from the likelihood-based *p* values for the same pathogens in Table 2 (e.g., coronavirus NL63, *p* = 0.050). The NL63 association was based on a single event in the oxygen group and should be regarded as hypothesis-generating rather than as a genuine protective effect. The median LOS was 4 days (IQR 2–6) and was longer in children who required oxygen (4 vs. 3 days, *p* < 0.001). RSV and human metapneumovirus (hMPV) were associated with the longest stays (median 5 days for both). Rehospitalization was infrequent (17 children, 3.9%) and did not differ by oxygen requirement (4.5% vs. 3.4%, *p* = 0.76); this outcome was underpowered and is reported descriptively only.

## 4. Discussion

We evaluated the association and predictive ability of widely available conventional host biomarkers, accepted danger signs of a severe course of ALRI, and radiologic findings for subsequent supplemental oxygen requirement in a retrospective cohort of children with a positive respiratory PCR panel. In our cohort of 439 children hospitalized with either an acute respiratory infection or a respiratory tract infection excluded as the cause of their presenting illness, the need for supplemental oxygen was strongly predicted by age, number of danger signs, and abnormal lung auscultation, but not by inflammatory markers. Throughout this study, disease severity was operationalized as the subsequent need for supplemental oxygen; although clinically meaningful, this outcome is not fully synonymous with severe ALRI and is closely linked to the clinical decision to initiate oxygen therapy.

The lack of a significant association between CRP, PCT, and WBC and supplemental oxygen requirement in our study is consistent with the prevailing view in the pediatric literature. Current data indicate that classic inflammatory biomarkers have only limited ability to distinguish between mild and severe courses of CAP in children. They may have predictive value in identifying the most severe complications, such as empyema or sepsis; however, these complications occur relatively rarely [20,37]. At the same time, it is emphasized that inflammatory biomarkers should not be used alone to assess disease severity but should always be interpreted in the context of clinical evaluation, a finding also confirmed by our study [21,37].

Regarding the NLR, it showed a statistically significant association with oxygen requirement in the univariable analysis; however, this association was attenuated after adjustment for other clinical factors in the multivariable model. This suggests that NLR may be a useful marker, given its correlation with other indicators of disease severity and clinical presentation. Current data indicate that elevated NLR values are associated with a more severe course of the disease, longer hospital stays, and an increased risk of admission to the intensive care unit [24,38,39]. Among the routinely measured biomarkers, the NLR has one of the highest predictive values for adverse clinical outcomes in children with pneumonia [24]. It should be emphasized, however, that optimal threshold values for the NLR have not yet been clearly established, and most of the available data come from East Asian populations, which limits the generalizability of these results and highlights the need for additional confirmation in other populations [38,40].

Consistent with previous literature, we have found that clinical assessment is significant for identifying patients with oxygen requirements. We showed that the number of danger signs, age, and abnormal lung auscultation, which are obtainable at the first clinical assessment without laboratory delay, can help predict the course of the disease. These results support the primacy of a structured and validated clinical assessment over laboratory investigations for identifying children at risk of respiratory compromise. This is consistent with previous evidence that clinical signs of respiratory distress are the strongest bedside predictors of hypoxemia in children and with contemporary multinational efforts to develop clinical severity scores for pediatric pneumonia [18,19,41]. Taken together, the principal clinical message of our study is that readily available clinical information, particularly respiratory examination findings, the cumulative number of danger signs, and age, was more informative than imaging or conventional inflammatory biomarkers for identifying children who subsequently required supplemental oxygen. This is especially relevant in emergency departments and general pediatric wards, where extensive or rapid diagnostic testing may not be immediately available.

Furthermore, the cumulative number of clinical danger signs at presentation proved to be a solid predictor of subsequent supplemental oxygen requirement, with greater numbers of danger signs consistently associated with worse clinical outcomes. Large multinational studies have confirmed that hypoxemia, chest retractions, and tachypnoea are the strongest clinical predictors of severity in pediatric pneumonia [19,42].

In univariate analysis, several imaging variables (e.g., lung consolidation on ultrasound, inflammatory changes on chest X-ray) were statistically significant. However, none retained significance in the multivariate model, suggesting that imaging may not add independent predictive value beyond clinical assessment. This is consistent with available data indicating that radiological changes, while associated with disease severity in univariate analyses, lose statistical significance when clinical variables are considered [19,43].

Among the 23 pathogens included in the panel, RSV showed the most consistent association with supplemental oxygen requirement and LOS. RSV-positive children required supplemental oxygen almost twice as often as RSV-negative children (60.3% versus 36.9%; OR 2.59, 95% CI 1.53–4.40) and had the longest median hospital stay in the cohort together with hMPV. This is consistent with the established role of RSV as an important viral cause of severe respiratory disease in young children and with burden estimates that underpin current immunoprophylaxis programs [5,44]. Two features of our data suggest that this association was not explained solely by the cohort’s age distribution. First, RSV was concentrated in the youngest children—44 of 68 detections (64.7%) occurred below 24 months—that is, in the age group with the lowest overall oxygen requirement in our cohort (28.6%). Second, RSV remained positively associated with oxygen requirement in the multivariable model, although the confidence interval crossed the null. The attenuation after adjustment may reflect the close relationship between RSV infection and clinical features of respiratory disease, including respiratory distress and abnormal lung auscultation. The practical implication is that a positive RSV result may add limited information to a bedside assessment that has already documented respiratory distress and abnormal auscultation, but may identify at admission a group with a higher probability of subsequently requiring oxygen and a longer hospital stay. This may support anticipatory planning of monitoring and bed capacity and is consistent with the rationale for maternal and monoclonal immunoprophylaxis programs.

A recent multicenter study (PERN) confirmed that clinical parameters remain the strongest predictors of disease severity, whereas imaging findings play only a supplementary role [19]. Additional evidence supporting this concept may encourage a more judicious use of imaging, particularly chest radiography, helping to avoid unnecessary examinations when they are unlikely to influence clinical decision-making. Interestingly, older age (>60 months) emerged as an independent predictor of oxygen requirement in our cohort, which contrasts with the prevailing evidence identifying younger age, particularly infancy below 6 months, as one of the strongest risk factors for severe pneumonia and hypoxemia [5,44,45].

One possible explanation for this phenomenon is that older children may remain under outpatient or home care for a longer period before being referred to the hospital, whereas younger children are sometimes admitted earlier despite a mild clinical presentation. This approach may stem from greater caution when caring for younger patients, whose clinical status can be more difficult to assess and whose threshold for hospital admission is often lower. Furthermore, decisions regarding hospital admission made in the emergency department can be influenced by the physician’s experience and often unfounded concerns about the risk of rapid clinical deterioration in younger children. An additional contributing factor may be the presence of asthma and recurrent viral-induced wheeze in older children, which could not be characterized in detail in our retrospective dataset. A stratified analysis by a history of comorbid asthma or wheeze was not performed because such an analysis could have resulted in small subgroups and unstable estimates; this remains an important question for future prospective studies. Importantly, this age association should not be interpreted as evidence that infants or younger children are intrinsically at lower risk of clinically important respiratory deterioration; rather, it most likely reflects center-specific admission thresholds, referral patterns, case mix, and the exclusion of critically ill children transferred to higher-level centers.

Our study has several important strengths. First, it included a relatively large cohort of children managed at a single pediatric center, assuring a homogeneous diagnostic and therapeutic approach, including consistent interpretation of imaging studies. Second, the primary outcome—requirement for supplemental oxygen—was clearly defined and ascertained across the entire cohort, providing a reproducible and clinically meaningful endpoint. We emphasize, however, that this endpoint reflects clinically important respiratory compromise rather than the full spectrum of ALRI severity, and that the decision to initiate oxygen therapy necessarily involves clinical judgment and may vary across institutions and clinicians; these considerations should be taken into account when interpreting the results. Third, all enrolled patients underwent the same multiplex PCR panel for 23 respiratory pathogens, affording comprehensive and uniform microbiological assessment and helping to reduce—though not eliminate—selection bias related to pathogen testing, as patients without available PCR results were excluded from the study.

Our study also has several limitations. First, this was a retrospective single-center study that relied on the accuracy and completeness of medical records and data extraction. Furthermore, the findings reflect the clinical practice of a single institution, which may limit their generalizability. External validation in multicenter cohorts is therefore warranted. Second, the retrospective verification of the diagnosis carries an inherent risk of disease misclassification. Third, we were unable to include children who presented to the emergency department with severe disease and were transferred to higher-level referral centers before hospital admission, possibly causing selection bias by excluding the most critically ill patients from the study cohort. Fourth, the primary outcome—requirement for supplemental oxygen—was assessed throughout the hospitalization rather than at admission, whereas predictor variables were recorded at presentation. Fifth, using supplemental oxygen requirement as the sole analyzed outcome limits direct comparison with the full composite severity classification; a sensitivity analysis using the composite outcome was not performed, as the infrequency of several individual components could have yielded unstable estimates. Sixth, procalcitonin measurements were unavailable for more than half of the study population because PCT testing was not routinely performed at our institution. This limited the assessment of its potential prognostic value in univariable analysis and precluded its inclusion in the multivariable model. Consequently, no conclusions regarding the independent prognostic value of PCT can be drawn from this study. Seventh, although oxygen saturation was assessed at hospital admission and the primary outcome reflected the requirement for supplemental oxygen at any time during hospitalization, oxygen saturation is an important determinant of the clinical decision to initiate oxygen therapy. Therefore, the observed association between low oxygen saturation and oxygen requirement should be interpreted with caution and not as evidence of a completely independent prognostic relationship. This association is further limited by incomplete documentation of key clinical variables: oxygen saturation was documented in only 230/439 children (~52%), while respiratory rate and maximum temperature were recorded in ~28% and ~66% of children, respectively. Such missingness may not have been completely random and may have reflected the child’s clinical condition at presentation; therefore, ascertainment bias cannot be excluded. A formal missing-data pattern analysis was beyond the scope of this retrospective study but represents an important direction for future prospective research. The multivariable model was based on complete-case data; although only a small proportion of patients were excluded, a potential selection effect cannot be entirely ruled out. A formal comparison of included versus excluded patients and multiple imputation were likewise beyond the scope of this retrospective study and represent, alongside the above, important directions for future prospective research. Eighth, given the exploratory nature of the study, a large number of univariable associations were tested without correction for multiple comparisons; borderline associations based on few events (e.g., Coronavirus NL63, rhonchi) should therefore be regarded as hypothesis-generating and interpreted with caution, as they may reflect chance findings. Ninth, the unit of analysis was the hospitalization episode rather than the individual child. 22 of the 439 episodes represented repeat admissions (20 children with >1 hospitalization), which may introduce a degree of within-patient correlation and potential non-independence of observations. Given this very small proportion (22/439, 5.0%), any resulting effect on the model estimates is likely to be negligible.

## 5. Conclusions

In this study, clinical factors, specifically the total number of danger signs, abnormal lung auscultation findings, and age, emerged as the strongest predictors of subsequent supplemental oxygen requirement, used here as a marker of disease severity. Conversely, routinely measured inflammatory biomarkers, including CRP and WBC, did not demonstrate independent prognostic value in the multivariate analysis, suggesting that their role in assessing disease severity may be limited. Imaging findings showed a similar pattern and likewise failed to demonstrate significance in the multivariate analysis. Among the pathogens assessed, RSV was the single detection associated with a distinctly more severe course, being linked to almost twofold higher odds of supplemental oxygen requirement and, together with hMPV, the longest hospital stay in the cohort; multiplex PCR nevertheless added little prognostic information beyond RSV status once bedside findings were taken into account. These results show the paramount importance of structured clinical assessment for the early identification of children who subsequently require supplemental oxygen. Nevertheless, given the retrospective nature of the study and its single-center design, further prospective, multicenter studies are needed to validate these observations and develop integrated tools to assess the risk of severe disease.

## Figures and Tables

**Table 1:** Clinical characteristics according to age group.

Observed Characteristics Among Hospitalized Patients									
Age Group	N	Oxygen Therapy	RSV Detected	Pneumonia	Chronic Disease	Abnormal Auscultation	Danger Signs, Median	CRP, Median	NLR, Median
0–6 months	134	45 (33.6%)	23.9%	16.4%	8.2%	53.0%	1.0	2.4	0.48
6–12 months	41	10 (24.4%)	9.8%	7.3%	0.0%	39.0%	2.0	6.9	1.36
12–24 months	66	14 (21.2%)	15.2%	21.2%	3.0%	31.8%	2.0	14.8	1.68
24–60 months	98	32 (32.7%)	16.3%	23.5%	15.3%	35.1%	1.0	25.3	2.86
60–120 months	64	50 (78.1%)	7.8%	17.2%	34.4%	27.0%	2.0	31.5	4.40
>120 months	35	27 (77.1%)	2.9%	5.7%	25.7%	14.3%	1.0	2.8	3.18

Age data were unavailable for one patient; therefore, the total number of patients included in age group categories is 438.

**Table 2:** Univariable logistic regression analyses.

Associations Between Candidate Predictors and Oxygen Therapy Requirement					
Predictor	N	Events	OR (95% CI)	*p *-Value	AUC
**Demographics**					
Age, months	438	178	1.02 (1.01–1.02)	<0.001	0.676
Season of infection: Autumn vs. reference	439	178	1.03 (0.63–1.66)	0.916	0.556
Season of infection: Spring vs. reference	439	178	0.63 (0.39–1.03)	0.065	0.556
Season of infection: Summer vs. reference	439	178	0.65 (0.31–1.33)	0.237	0.556
Sex: Male vs. reference	438	178	1.22 (0.83–1.78)	0.318	0.524
**Danger signs**					
Accessory respiratory muscle use: Yes vs. reference	438	178	4.31 (2.45–7.57)	<0.001	0.594
At least one danger sign: Yes vs. reference	439	178	1.71 (0.94–3.13)	0.080	0.529
Audible wheezing without auscultation: Yes vs. reference	439	178	1.86 (0.49–7.01)	0.361	0.506
Number of danger signs	439	178	1.59 (1.35–1.88)	<0.001	0.649
Dyspnea: Yes vs. reference	437	178	4.52 (2.75–7.44)	<0.001	0.623
Reduced appetite, feeding refusal or feeding difficulty: Yes vs. reference	439	178	1.02 (0.63–1.66)	0.932	0.502
Fever at admission: Yes vs. reference	438	178	0.56 (0.37–0.84)	0.005	0.564
Grunting: Yes vs. reference	439	178	Not estimable		
Peripheral oxygen saturation ≤92%: Yes vs. reference	230	120	8.83 (3.00–26.02)	<0.001	0.607
Tachypnea: Yes vs. reference	439	178	6.23 (3.36–11.54)	<0.001	0.609
Vomiting: Yes vs. reference	439	178	1.14 (0.73–1.79)	0.570	0.512
**Other clinical characteristics**					
Abnormal lung auscultation: Yes vs. reference	437	178	3.74 (2.49–5.62)	<0.001	0.653
Chronic disease or suspected chronic condition: Yes vs. reference	439	178	2.31 (1.33–4.02)	0.003	0.550
Cough: Yes vs. reference	438	178	1.92 (1.29–2.84)	0.001	0.579
Crackles: Yes vs. reference	437	178	6.44 (3.54–11.72)	<0.001	0.618
Gastrointestinal symptoms: Yes vs. reference	437	177	1.15 (0.78–1.69)	0.476	0.517
Maximum body temperature, °C	288	105	0.67 (0.51–0.88)	0.004	0.607
Pneumonia: Yes vs. reference	439	178	3.20 (1.91–5.37)	<0.001	0.583
Duration of prodromal symptoms, days	411	169	1.03 (0.98–1.09)	0.234	0.537
Prolonged expiration: Yes vs. reference	439	178	4.16 (2.20–7.86)	<0.001	0.572
Respiratory rate, breaths/min	121	80	1.02 (0.99–1.04)	0.229	0.556
Rhinitis: Yes vs. reference	438	178	1.36 (0.93–1.99)	0.118	0.538
Rhonchi: Yes vs. reference	437	178	1.76 (1.11–2.79)	0.017	0.548
Wheezing on auscultation: Yes vs. reference	437	178	2.69 (1.54–4.72)	<0.001	0.560
**Laboratory markers**					
CRP, mg/L	437	176	1.00 (1.00–1.00)	0.975	0.526
Lymphocyte count	433	175	0.94 (0.88–1.00)	0.056	0.581
Neutrophil count	433	175	1.03 (1.00–1.06)	0.085	0.546
NLR	433	175	1.12 (1.06–1.19)	<0.001	0.586
PCT	208	67	1.02 (0.95–1.11)	0.532	0.518
WBC	438	177	0.99 (0.97–1.02)	0.723	0.509
**Etiology and pathogen burden**					
Coinfection (≥2 pathogens): Yes vs. reference	439	178	0.91 (0.59–1.42)	0.686	0.509
Etiology: Bacterial pneumonia vs. reference	439	178	4.33 (0.61–30.57)	0.141	0.590
Etiology: Bacterial superinfection of viral pneumonia vs. reference	439	178	3.31 (1.06–10.41)	0.040	0.590
Etiology: Other bacterial superinfection of a viral respiratory tract infection vs. reference	439	178	0.77 (0.23–2.52)	0.664	0.590
Etiology: Other disease vs. reference	439	178	1.28 (0.43–3.78)	0.654	0.590
Etiology: Pertussis vs. reference	439	178	2.17 (0.12–40.81)	0.606	0.590
Etiology: Respiratory viral infection vs. reference	439	178	1.51 (0.56–4.11)	0.417	0.590
Etiology: Uncertain etiology vs. reference	439	178	Not estimable		
Number of detected pathogens	439	178	1.06 (0.83–1.35)	0.644	0.525
Respiratory tract infection: Yes vs. reference	439	178	1.37 (0.85–2.20)	0.194	0.526
**Pathogens**					
Adenovirus	439	178	0.50 (0.29–0.84)	0.009	0.550
Bordetella parapertussis (IS1001)	439	178	Not estimable		
Coronavirus 229E	439	178	Not estimable		
Coronavirus HKU1	439	178	Not estimable		
Coronavirus NL63	439	178	0.13 (0.02–1.00)	0.050	0.518
Coronavirus OC43	439	178	0.93 (0.35–2.45)	0.884	0.501
hMPV	439	178	1.72 (0.87–3.40)	0.122	0.521
Human Rhinovirus/Enterovirus	439	178	1.09 (0.73–1.60)	0.679	0.510
Influenza A	439	178	1.68 (0.64–4.45)	0.293	0.510
Influenza B	439	178	1.79 (0.54–5.94)	0.344	0.507
Parainfluenza Virus 1	439	178	0.81 (0.27–2.46)	0.709	0.503
Parainfluenza Virus 2	439	178	Not estimable		
Parainfluenza Virus 3	439	178	1.74 (0.57–5.27)	0.327	0.508
Parainfluenza Virus 4	439	178	1.48 (0.42–5.19)	0.540	0.504
RSV	439	178	2.59 (1.53–4.40)	<0.001	0.563
Severe Acute Respiratory Syndrome Coronavirus 2 (SARS-CoV-2)	439	178	0.27 (0.10–0.73)	0.009	0.534
**Chest X-ray findings**					
Hilar enlargement	431	176	5.91 (0.65–53.30)	0.114	0.509
Increased or thickened bronchial markings	431	176	1.69 (0.60–4.74)	0.321	0.509
Increased vascular markings	431	176	0.29 (0.03–2.47)	0.255	0.507
Inflammatory or parenchymal chest X-ray changes	431	176	1.64 (1.02–2.64)	0.040	0.541
Normal chest X-ray	431	176	0.97 (0.64–1.45)	0.868	0.504
Chest X-ray performed: Yes vs. reference	431	176	1.46 (0.98–2.17)	0.059	0.545
Pleural effusion on chest X-ray	431	176	4.53 (1.21–16.97)	0.025	0.520
Reduced aeration or atelectasis	431	176	1.76 (0.53–5.87)	0.355	0.507
**Lung ultrasound findings**					
Consolidation on lung ultrasound	438	178	4.77 (2.18–10.45)	<0.001	0.556
Interstitial changes on lung ultrasound	438	178	2.70 (1.25–5.83)	0.011	0.532
Normal lung ultrasound	438	178	1.05 (0.45–2.41)	0.916	0.501
Lung ultrasound performed: Yes vs. reference	438	178	2.22 (1.32–3.73)	0.003	0.555
Pleural effusion on lung ultrasound	438	178	8.50 (1.86–38.82)	0.006	0.527
Odds ratios (ORs), confidence intervals (CIs)	Odds ratios (ORs), confidence intervals (CIs)	Odds ratios (ORs), confidence intervals (CIs)	Odds ratios (ORs), confidence intervals (CIs)	Odds ratios (ORs), confidence intervals (CIs)	Odds ratios (ORs), confidence intervals (CIs)

Odds ratios (ORs) and 95% confidence intervals (CIs) were estimated using univariable logistic regression. Bias-reduced logistic regression was used when a zero cell suggested complete or quasi-complete separation. AUC denotes the area under the receiver operating characteristic curve for each univariable model. Pathogen-specific models were estimated only for pathogens detected in at least 5 patients. Results are reported as Not estimable when this threshold was not met or when the fitted model did not converge. These unadjusted analyses were exploratory and were not used as an automatic variable-selection rule.

**Table 3:** Pathogens detected by multiplex PCR. Frequency of detected pathogens according to oxygen therapy requirement.

		Primary Outcome	Primary Outcome	
	Overall N = 439	No Oxygen Therapy N = 261	Oxygen Therapy N = 178	*p*-Value
Human Rhinovirus/Enterovirus	170 (38.7%)	99 (37.9%)	71 (39.9%)	0.691
Adenovirus	83 (18.9%)	60 (23.0%)	23 (12.9%)	0.009
RSV	68 (15.5%)	27 (10.3%)	41 (23.0%)	<0.001
hMPV	36 (8.2%)	17 (6.5%)	19 (10.7%)	0.156
Severe Acute Respiratory Syndrome Coronavirus 2 (SARS-CoV-2)	30 (6.8%)	25 (9.6%)	5 (2.8%)	0.006
Coronavirus OC43	18 (4.1%)	11 (4.2%)	7 (3.9%)	>0.999
Influenza A	17 (3.9%)	8 (3.1%)	9 (5.1%)	0.320
Parainfluenza Virus 1	14 (3.2%)	9 (3.4%)	5 (2.8%)	0.788
Parainfluenza Virus 3	13 (3.0%)	6 (2.3%)	7 (3.9%)	0.393
Coronavirus NL63	12 (2.7%)	11 (4.2%)	1 (0.6%)	0.032
Influenza B	11 (2.5%)	5 (1.9%)	6 (3.4%)	0.365
Parainfluenza Virus 4	10 (2.3%)	5 (1.9%)	5 (2.8%)	0.535
Bordetella parapertussis (IS1001)	4 (0.9%)	2 (0.8%)	2 (1.1%)	>0.999
Coronavirus 229E	2 (0.5%)	2 (0.8%)	0 (0.0%)	0.517
Coronavirus HKU1	2 (0.5%)	0 (0.0%)	2 (1.1%)	0.164
Parainfluenza Virus 2	1 (0.2%)	1 (0.4%)	0 (0.0%)	>0.999

## Data Availability

The original contributions presented in this study are included in the article/Supplementary Material. Further inquiries can be directed to the corresponding author.

## Supplementary Materials

The following supporting information can be downloaded at: https://www.mdpi.com/article/10.3390/microorganisms14091926/s1, Table S1: Overview of current severity classification criteria for pediatric community-acquired pneumonia.

## Abbreviations

The following abbreviations are used in this manuscript:

ALRI	Acute lower respiratory infection
RSV	Respiratory syncytial virus
CAP	Community-acquired pneumonia
PSI	Pneumonia Severity Index
CURB-65	Confusion, Urea, Respiratory Rate, Blood Pressure, and Age ≥65 years
CRP	C-reactive protein
PCT	Procalcitonin
WBC	White blood cell
NLR	Neutrophil-to-lymphocyte ratio
sTREM-1	Soluble triggering receptor expressed on myeloid cells-1
PCR	Polymerase chain reaction
IV	Intravenous
ORs	Odds ratios
CIs	Confidence intervals
LOS	Length of Stay
