# Supplementary material for: Early Clinical and Inflammatory Predictors of Supplemental Oxygen Requirement in Children Hospitalized with Acute Lower Respiratory Infection: A Multivariable Analysis
Source: Microorganisms. 2026 Sep 1;14(9):1926. doi: 10.3390/microorganisms14091926 (PMC13609486; doi:10.3390/microorganisms14091926)
Supplement: Supplementary file 1 [file microorganisms-14-01926-s001.zip › microorganisms-4475257-supplementary.pdf]

Table S1. Overview of current severity classification criteria for pediatric community-acquired pneumonia.

Feature	WHO/IMCI (2014)	PIDS/IDSA (2011)	BTS
**Target population**	Children 2–59 months; low- and middle-income countries	Children >3 months; high-income countries	Children; high-income countries
**Severity categories**	Pneumonia (non-severe) vs. severe pneumonia	Simple vs. complicated; major/minor criteria for ICU admission	Severe pneumonia based on clinical criteria and complications
**Key severity criteria**	Cough/dyspnea + any general danger sign (inability to feed, vomiting, convulsions, lethargy, stridor)	Major: invasive ventilation, refractory shock, NIPPV, hypoxemia; Minor: tachypnea, apnea, increased work of breathing, multilobar infiltrates, altered mental status	High fever + severe general symptoms, significant dyspnea, cyanosis; ± respiratory/heart failure, toxic encephalopathy, empyema, sepsis
